# Nature’s economy as blueprint: regional overlap & block-based algorithm for protein design

**DOI:** 10.3389/fbinf.2026.1746135

**Published:** 2026-05-21

**Authors:** Héctor Alexis Retana-Fonseca, Roberto Damián-Tentle, Luis A. Pineda, Myriam M. Altamirano-Bustamante

**Affiliations:** 1 Unidad de Investigación en Enfermedades Metabólicas, Centro Médico Nacional Siglo XXI, Instituto Mexicano del Seguro Social, Ciudad de México, Mexico; 2 Instituto de Investigaciones en Matemáticas Aplicadas y en Sistemas, Universidad Nacional Autónoma de México, México, Mexico

**Keywords:** bioinformatics, Hsp70, motif detection, protein design, protein disulfide isomerase, protein evolution, rule-based algorithm, structural conservation

## Abstract

**Introduction:**

Nature has optimized protein design through modular recombination of conserved and variable domains, allowing rapid adaptation with minimal energetic cost. Inspired by this principle, we present ROB-Fold, a deterministic rule-based algorithm that emulates natural evolution to identify conserved structural scaffolds across protein families.

**Methods:**

Using experimentally validated and AI-predicted models from AlphaFold and the Protein Data Bank, ROB-Fold establishes motif centered analyses anchored on biologically meaningful patterns, into study cases, that can be extended for any protein family, such as CXXC in protein disulfide isomerases (PDIs) and EEVD in HSP70 chaperones. Around each anchor, bidirectional expansions are generated to detect recurrent sequence and structural elements. The algorithm translates residue frequencies into Chou–Fasman structural equivalence classes, projects consensus patterns onto secondary structure, and quantifies geometric persistence via Root Mean Square Deviation (RMSD).

**Results:**

Application to the PDI family revealed a conserved β–α–β–α–β core centered on the CXXC catalytic motif, delineating the thioredoxin-like scaffold responsible for redox activity. Similarly, analysis of the HSP70 family identified a recurrent “GPXVEEVD” pattern defining the nucleotide-binding domain, with conserved α-helical arrangements surrounding the EEVD motif. Across both cases, conserved regions exhibited RMSD values below 15 Å, confirming structural persistence and validating the cross-family generalization of the rule-based logic.

**Discussion:**

These findings demonstrate that explicit structural rules can recover evolutionary invariants typically detected by stochastic models while maintaining interpretability and computational efficiency. ROB-Fold thus provides a transparent and biologically grounded framework for identifying natural scaffolds, enabling a guide for the rational design of novel proteins.

## 1 Introduction

Nature’s protein landscape show us a conspicuous and economical way to allow the rapid generation of novel proteins by a combinatorial design rules in the evolution (Liang and Nie, 2020; Villalobos-Alva et al., 2022). The challenge is understanding this rules of construction. In this work we aim to mimicking natural evolution in order to identify the conserved features scaffold and the variable features-one that leverages the inherent modularity of protein domains-to allow the rapid construction of lego-structural modules that complete the puzzle in the target proteins (Yamada and Kinoshita, 2018; Qin et al., 2020; Siedhoff et al., 2020). By reverse-engineering the principles that govern natural protein evolution Mixing and matching the lego-structural modules we be able to develop novel scaffold that non-existent in the world using simplifying and economical route that Nature has already evolved, it is possible to develop new methods that streamline the design process while maintaining functional efficacy while minimizing environmental impact (Hey et al., 2019; Senior et al., 2020; AlQuraishi, 2021).

In this study, an innovative AI-bioinformatics toolkit designed to facilitate the sustainable engineering of novel proteins is proposed. The approach is based on the principle of identifying and utilizing conserved scaffolds, while allowing for controlled variation in specific regions to introduce desired functionalities. This methodology mirrors the way nature fine-tunes protein functions through post-translational modifications and domain interactions, particularly through protein-disulfide isomerase (PDI) patterns. PDI plays a crucial role in protein folding and stability and understanding its evolutionary patterns can provide key insights for rational protein design (Kroth et al., 2012; Al-Gharabli et al., 2018; Dara et al., 2019). By integrating AI-driven algorithms with bioinformatic analysis of conserved and variable regions in proteins, we can develop a toolkit that allows for the precise and efficient design of novel proteins (Senior et al., 2020; Villalobos-Alva et al., 2022). This toolkit aims to optimize computational resources by focusing on biologically validated structural patterns, significantly reducing the time and energy required for new protein designs (Raveh et al., 2007; Al-Gharabli et al., 2018; Yang et al., 2020).

(Kayum et al., 2017; Moretti et al., 2017). This bioinspired strategy opens new avenues for designing proteins with targeted applications, paving the way for more ecologically responsible and technologically advanced solutions in the field of protein folding-design and evolution.

An immediate application of this approach could lie in the field of protein-conformational disorders (such as Alzheimer’s disease, diabetes, and cancer), which are catastrophic owing to the high economic and social burden on families and healthcare institutions. Two common features of these disorders are, first, that they are diagnosed very late—by which time the target organ is already about 60% destroyed (the pancreas in the case of diabetes, the brain in the case of Alzheimer’s disease)—and second, that to date there is no effective treatment. The development of novel proteins—ones not found in nature—with foldase and chaperone-like properties is an urgent necessity to generate potential therapeutic agents.

In this paper we choice two proteins family as scaffold to test Rod-fold algorithm. Case A: Protein disulfide isomerase family and Case B: Hsp70 family.

### 1.1 Case A

PDI is a protein found in the endoplasmic reticulum of eukaryotic cells and has a molecular weight of approximately 55 kDa. Structurally, PDI is composed of several domains arranged in the sequence abb’a’c, where the a and a’ domains are crucial for its enzymatic function. These domains each contain an active site with the sequence CGHC that alternates between a reduced (thiols) and an oxidized (disulfide) state. This redox cycling allows PDI to facilitate various processes involving disulfide bonds in other proteins, such as creating, rearranging, or breaking these bonds (Westphal et al., n.d.). For PDI to successfully transfer disulfide bonds to a protein, it must be in its oxidized form. Conversely, when proteins are incorrectly folded, often having incorrect disulfide linkages, PDI inverses to correct these errors (Kim, n.d.). It achieves this by catalyzing isomerization reactions; it breaks the improper disulfide bonds and assists in their reformation into correct native disulfide arrangement, ensuring proper protein folding and function.

### 1.2 Case B

Heat shock proteins (HSPs) constitute a group of highly conserved polypeptides present in nearly all subcellular compartments. These proteins are categorized according to their molecular mass and act as molecular chaperones that preserve protein stability and cellular integrity. Among them, the HSP70 family represents one of the most widespread classes, being detected across multiple cellular locations, including the cytosol, membranes, endoplasmic reticulum, mitochondria, and chloroplasts, as well as in the extracellular environment. Homologs of HSP70 are also found in bacteria and certain archaea, where they perform specialized organelle- or tissue specific roles (Sharma et al., 2009; Kampinga and Craig, 2010). In many biological contexts, HSP70 members exhibit functional redundancy, and their chaperone activity relies on ATP-driven cycles that regulate the binding and release of exposed hydrophobic segments in substrate proteins (Gribaldo et al., 1999; Sharma and Masison, 2009).

## 2 Methods

### 2.1 Algorithmic overview

ROB-Fold is a deterministic, rule-based algorithm designed to identify conserved structural scaffolds through hierarchical inference across the three structural layers of proteins: primary sequence, secondary topology, and three-dimensional conformation.

The system integrates explicit biological reasoning, encoded as computational rules, with structural data from experimentally models, like AlphaFold. Unlike stochastic or data intensive methods, ROB-Fold operates through logical propagation rather than statistical learning, yielding results that are interpretable, reproducible, and computationally efficient.

**FIGURE 1:**
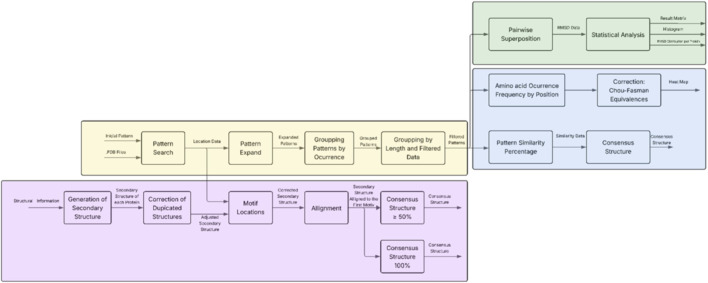
General diagram of the algorithm for structural conservation and variability analysis. The flow is organized into four different modules that interact sequentially. The main or general processing module (yellow) is responsible for receiving, standardizing, and preparing the information needed to feed the specific modules. The primary structure module (blue) is responsible for analysing amino acid residue sequences. The secondary structure module (purple) identifies and compares elements such as α helices and β sheets. Finally, the three-dimensional structure module (green) performs spatial structural analysis using three-dimensional alignments.

The algorithm is implemented in Python as a modular pipeline, shown in Figure 1, composed of a backend engine responsible for preprocessing, expansion, and rule execution and a structure-handling module for extracting and aligning topological and geometric information.

### 2.2 General processing

The analytical workflow begins with normalization of input data. For each protein, the sequence and 3D coordinates are parsed and standardized. Using user defined anchor motifs, identified by regular expressions (e.g., CXXC in PDIs or EEVD in HSP70s), ROB-Fold establishes structural reference points that serve as origins for analysis.

From each anchor, bidirectional expansions are performed toward the N- and C-termini in discrete increments of ten residues up to 200 based on typical domain sizes, conserved fold neighborhoods and motif-centered functional regions. This symmetric logic ensures unbiased contextual exploration around the motif. When sequence boundaries are exceeded, a neutral padding residue (“X”) is introduced, maintaining dimensional homogeneity and permitting cross family comparisons among proteins of differing lengths.

Expansion sets are grouped by length and filtered to guarantee phylogenetic representativeness; each length category retains at least one sequence from every taxonomic subgroup. This step produces standardized, length homogeneous datasets that form the foundation for subsequent inference layers.

### 2.3 Fold one: primary structure

Fold One identifies residue-level conservation patterns by constructing positional frequency matrices for each expansion length. These matrices quantify amino acid recurrence and positional variability relative to the anchor motif.

To transcend pure sequence comparison, each residue is further translated into a structural equivalence class following the Chou–Fasman principle, which groups amino acids according to their secondary structure propensities (α-helix, β-sheet, turn).

This equivalence allows ROB-Fold to detect structural conservation even where sequence divergence occurs. Highly conserved positions are incorporated into consensus strings, while variable sites are labeled “X,” denoting functionally tolerant or adaptive positions. The resulting consensus captures the evolutionary fingerprint of the family’s structural nucleus.

### 2.4 Fold two: secondary structure

Fold Two projects the primary consensus into the secondary structure dimension. Secondary assignments are derived using KSDSSP from UCSF Chimera. Each secondary element (helix, strand, coil) is aligned relative to the anchor motif to enable cross-family comparison of topological arrangements.

Through a weighted overlap scheme, the algorithm merges homologous expansions, identifying regions with partial (≥50%) or complete (100%) recurrence. This rule distinguishes flexible or functionally divergent segments from highly constrained structural cores. The resulting consensus topology often converges on canonical folding patterns such as β–α–β–α–β architectures, characteristic of stable redox and chaperone domains.

### 2.5 Fold three: three-dimensional structure

Fold Three validates geometric conservation at the spatial level. For each candidate scaffold, Cα coordinates are extracted and pairwise Root Mean Square Deviation (RMSD) values computed across all homologs.

The resulting RMSD distribution defines a conservation radius, the expansion length within which most structures maintain deviations below 10–15 Å. Positions inside this radius are classified as structurally conserved; those outside are interpreted as flexible structures.

This step bridges sequence level conservation with structural persistence, confirming which motifs act as geometric scaffolds across the family.

### 2.6 Rule-based framework

ROB-Fold’s interpretability emerges from its explicit inference rules. Each rule, explained in Table 1, encodes a biological rationale and computational purpose, transforming empirical observation into structured logic.

**TABLE 1:** Description rules.

Rule	Biological rationale	Computational role
Anchor recognition	Conserved motifs act as nucleation points for folding and catalysis (e.g., CXXC, EEVD)	Defines reference coordinates for expansion and alignment
Symmetric expansion	Evolutionary conservation often radiates symmetrically from functional cores based on typical domain sizes, conserved fold neighborhoods and motif-centered functional regions	Ensures unbiased contextual sampling around anchors
Structural equivalence	Functionally equivalent residues may differ in sequence but share folding tendencies	Enables detection of hidden conservation through Chou–Fasman mapping
Alignment integration	Structural stability arises when secondary structure elements (α-helices, β-sheets) occupy equivalent topological positions across homologs	Evaluates positional recurrence of these elements and alignment them, distinguishing rigid structural nuclei from flexible or adaptive segments
Geometric validation	Evolutionarily conserved scaffolds maintain spatial persistence	Quantifies conservation radius via RMSD, validating structural cores

## 3 Results

### 3.1 Case A: identification of conserved scaffolds in the PDI family

Applying ROB-Fold to the Protein Disulfide Isomerase (PDI) family, described in Table 2, revealed a pronounced conservation gradient centered on the CXXC catalytic motif.

**TABLE 2:** Summary of UniProt derived PDI Family proteins.

Uniprot code	Name	Organism	3D structure source
*Q86IA3*	PDI	*Dictyostelium discoideum*	AlphaFoldv2
*P17967*	PDI1	*Saccharomyces cerevisiae*	PDB
*Q9XI01*	PDI2	*Arabidopsis thaliana*	AlphaFoldv2
*Q17770*	PDI3	*Caenorhabd itis elegans*	AlphaFoldv2
*Q9V438*	PDI4	*Drosophila melanogaster*	AlphaFoldv2
*P05307*	PDI5	*Bos Taurus*	AlphaFoldv2
*P38660*	PDI6	*Mesocricetus auratus*	AlphaFoldv2
*P38659*	PDI7	*Rattus norvegicus*	AlphaFoldv2
*D3Z6P0*	PDI8	*Mus musculus*	AlphaFoldv2
*Q4VIT4*	PDI9	*Chlorocebus aethiops*	AlphaFoldv2
*Q14554*	PDI10	*Homo sapiens*	AlphaFoldv2

**FIGURE 2:**
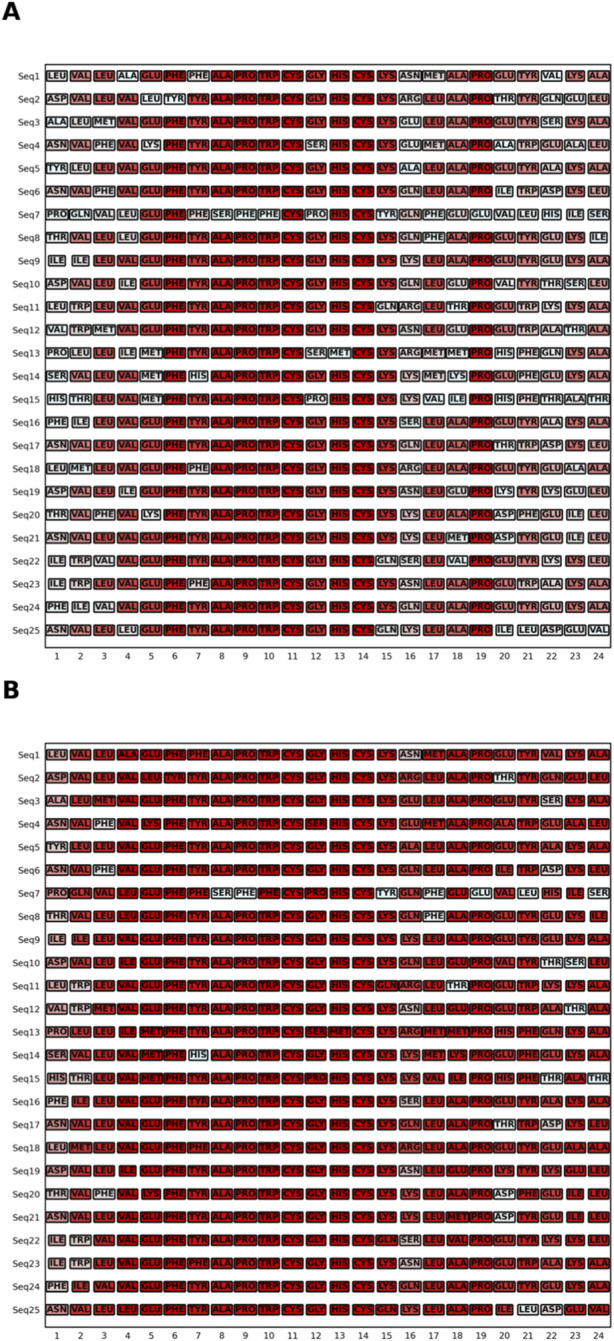
Frequency of residue occurrence in a PDI Family. **(A)** Heat map of amino acid residue occurrence frequency on aligned sequences of length twenty-four. Conserved regions are shown in deep red tones, while variable regions are shown in lighter tones. **(B)** Heat map based on grouping residues according to their structural equivalences (propensity to form helices, sheets, or turns) proposed by Chou-Fasman.

Primary sequence analyses showed residue fixation around the motif, while flanking regions exhibited variable (Figure 2A). Translation into Chou–Fasman equivalence classes reclassified several variable positions as structurally conserved, indicating persistence of folding tendencies despite sequence divergence (Figure 2B).

To quantify the degree of conservation during the expansion process, we calculated the percentage of Sequence Identity (SI) between homologous pairs of equal length. This was defined as Equation 1 where D represents the number of non-identical amino acids (Hamming distance) between the two sequences, and L denotes the total length of the analysed segment.

$$SI=100\times \left(1-\frac{D}{L}\;\right)$$ (1)

**FIGURE 3:**
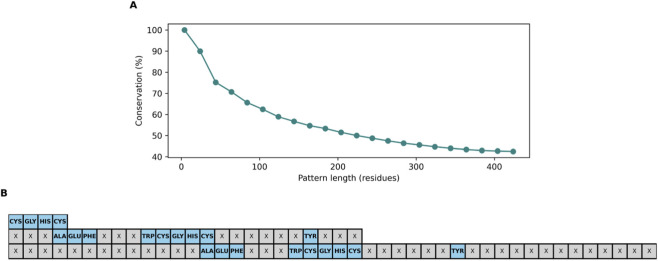
Structural conservation in a PDI Family. **(A)** Average structural conservation (%) as a function of pattern length. The average of similarity between patterns of the same length is shown. **(B)** Consensus sequence derived from phylogenetic analysis. Residues conserved at 100% are indicated in blue; variable residues are represented by “X.” The analysis covers patterns of up to 44 residues.

This allows us to understand how, as the patterns found expand, they become less similar (Figure 3). This behavior suggests that the areas of high conservation are those adjacent to the initial motif, which reinforces the idea that their immediate environment is key to their biological activity. The resulting consensus delineated a core “AEFXAPWCGHCKXXXPXYXXXXXXL” module that constitutes the functional nucleus of the thioredoxin like domain (Figure 3B).

#### 3.1.1 Topological and structural validation

**FIGURE 4:**
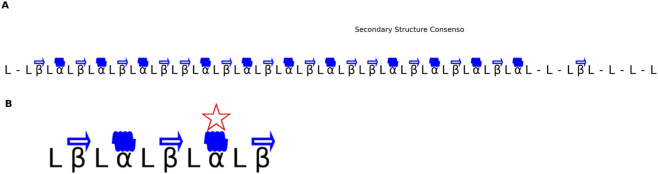
Consensus Secondary Structure in a PDI Family. **(A)** Consensus structure based on the appearance of the same type of secondary structure (alpha or beta) in at least 50% of proteins. **(B)** 100% conserved consensus structure, representing structural regions that are invariant across all proteins in the phylogenetic tree.

**FIGURE 5:**
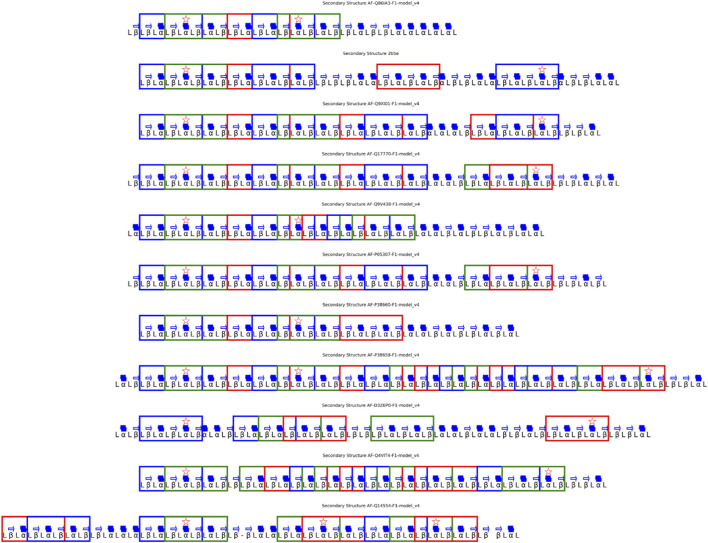
Representation of the β–α–β–α–β consensus pattern in a PDI Family. The location of the structural segments that are 100% conserved throughout proteins aligned at first motif. To avoid confusion in the case of overlapping patterns, four distinct colours were used to distinguish them.

Fold Two confirmed the recurrence of α-helices and β-sheets at equivalent topological positions across phylogenetically distant PDIs, yielding a reproducible β–α–β–α–β topology (Figure 4). This organization coincided with low RMSD regions overlapped with the enzyme’s active-site cavity, underscoring its catalytic and structural significance (Figure 5).

**FIGURE 6:**
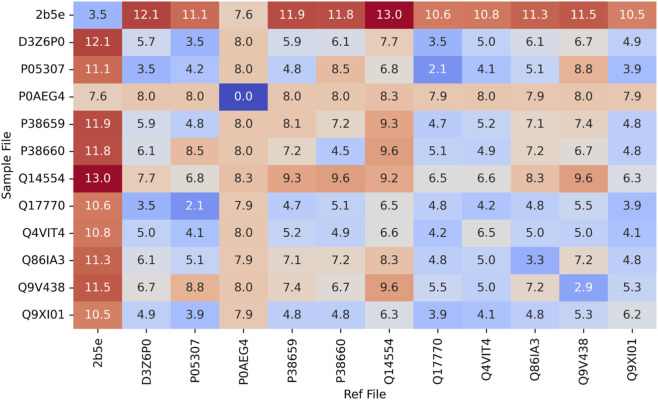
Mean RMSD Heatmap from a PDI Family. Heatmap showing the average Root Mean Square Deviation (RMSD) values across all pairwise structural comparisons among PDI homologs. Low RMSD regions (<15 Å) correspond to the conserved β–α–β–α–β core, delineating the empirical conservation radius and distinguishing stable scaffolds from flexible adaptive segments.

**FIGURE 7:**
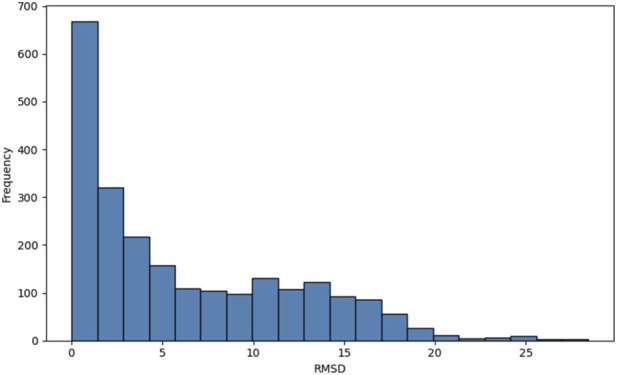
RMSD Frequency Histogram: This histogram captures the complete landscape of pairwise structural comparisons within the generated model.

Fold Three validation reinforced these observations: expansions up to 200 residues maintained mean RMSD values below 15 Å (Figure 6). To better understand structural heterogeneity, the distribution of all pairwise RMSD values was analysed (Figure 7). The distribution is predominantly unimodal and right skewed, with a median of 6.1498 Å and a standard deviation of ± 5.9669 Å.

### 3.2 Case B: cross-family application HSP70

**FIGURE 8:**
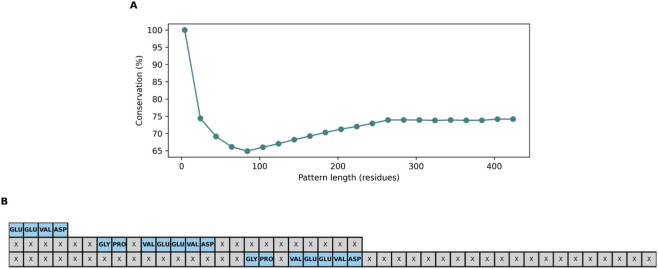
**(A)** Average structural conservation (%) as a function of pattern length. **(B)** Schematic motif alignment highlighting repeated amino acids.

To assess generality, ROB-Fold was applied to the HSP70 chaperone family, described in Table 3, using EEVD as the anchor motif. The algorithm identified a recurrent “GPXVEEVD” pattern demarcating the nucleotide-binding domain (NBD) (Figure 8).

**TABLE 3:** Summary of UniProt derived HSP70 proteins.

Uniprot code	Name	Organism	3D structure source
*P10591*	HSP70	*Saccharomyces cerevisiae*	AlphaFoldv2
*P11142*	HSP70-1	*Homo Sapiens*	AlphaFoldv2
*P22953*	HSP70-2	*Arabidopsis thaliana*	AlphaFoldv2
*P25840*	HSP70-3	*Chlamydomonas reinhardtii*	AlphaFoldv2
*P82910*	HSP70-4	*Drosophila melanogaster*	AlphaFoldv2
*Q00043*	HSP70-5	*Ajellomyces capsulatus*	AlphaFoldv2
*Q61696*	HSP70-6	*Mus musculus*	AlphaFoldv2

**FIGURE 9:**
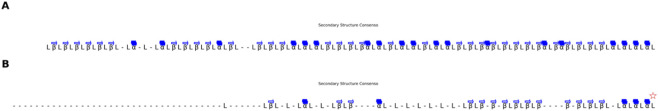
Consensus Secondary Structure in the HSP70 Family. **(A)** Consensus map showing the recurrence of α-helices and β-sheets in at least 50% of homologs, aligned relative to the EEVD anchor motif. Conserved elements delineate the structural nucleus of the nucleotide-binding domain (NBD), while variable regions correspond to flexible linker segments. **(B)** 100% conserved consensus structure, highlighting invariant regions that remain spatially aligned across all analyzed homologs, defining the topological core of the EEVD-centered scaffold.

**FIGURE 10:**
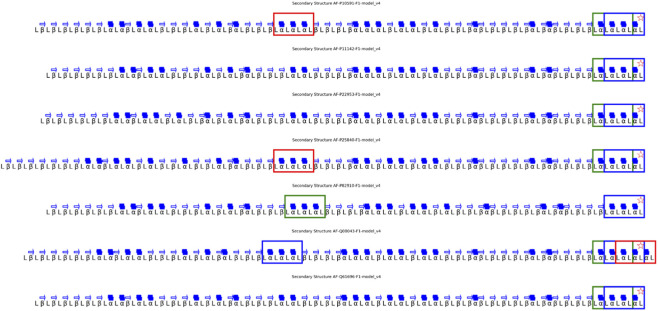
Structural Validation of the 100% Consensus Pattern in the HSP70 Family. Search and mapping of the α-α–α consensus pattern derived from the 100% conserved secondary structure across all homologs. Each detected pattern is represented along the aligned secondary structures, confirming recurrence of the consensus topology within the HSP70 family and validating the rule-based inference of ROB-Fold.

The consensus map, Figure 9, revealed recurrent α-α–α elements surrounding the EEVD motif, suggesting a structurally stable core within the nucleotide-binding domain (Figure 10).

**FIGURE 11:**
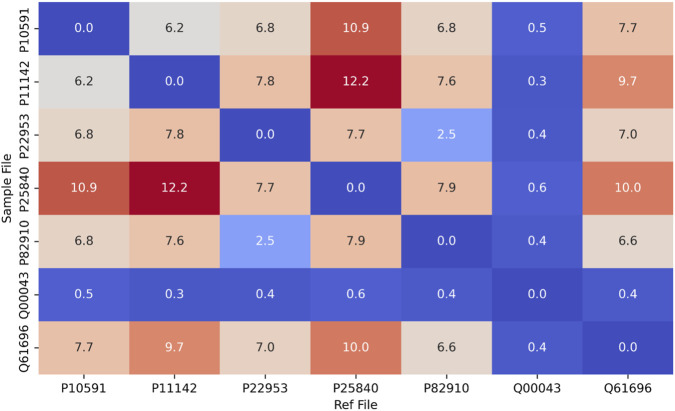
Mean RMSD Heatmap from a HSP70 Family. Heatmap representing the average Root Mean Square Deviation (RMSD) across pairwise comparisons of HSP70 homologs. Low RMSD clusters (<15 Å) align with the core of the NBD, confirming the geometric persistence of the EEVD centered scaffold and validating ROB-Fold’s generalization across structurally distinct chaperones.

The mean RMSD (<15 Å) across seven homologs was comparable to values from the PDI family, confirming that ROB-Fold’s inference logic generalizes across unrelated protein architectures (Figure 11).

### 3.3 Non-biological-anchor control analysis

To validate that the conserved scaffolds identified by ROB-Fold are intrinsically linked to biological functional centers and not a byproduct of global protein homology, we implemented a Non-Biological-Anchor Control Analysis (n = 50). This experiment functions as a structured null model designed to test the system discriminative power against non-biological positions.

#### 3.3.1 Stratified sampling for structurally matched null model construction

Unlike a simple random sampling, we employed a stratified sampling strategy to select synthetic anchors that share the same structural context as the biological motif. This means:

- The exact same amino acid composition is preserved: This avoids biases associated with residue type and controls the contribution of chemical composition.
- The sequence is randomly reordered (permutation): The specific biological organization of the anchor motif is destroyed, while maintaining only its composition.
- Distance from the original biological anchor to the N-terminal and C-terminal ends was calculated.
- Safety margin: Defined to restrict the selection of random anchors to an equivalent structural window. This ensures that the null model accounts for positional biases and protein length, fulfilling the criteria for a structurally matched control.

#### 3.3.2 Test and conservation decay

**FIGURE 12:**
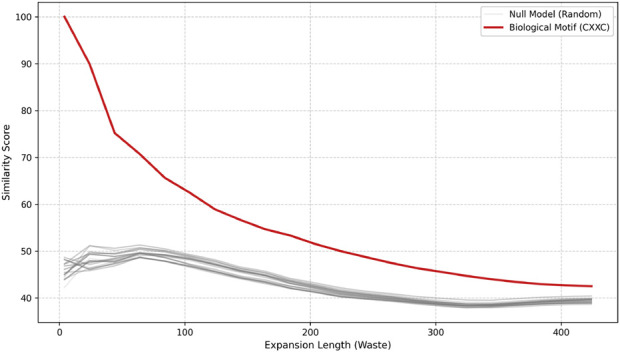
Structural Persistence Biological Anchors vs. Non-Biological Anchors (n = 50). Comparison between the biological motif (red) and the distribution of 50 non-biological anchors (grey). The sustained signal of the biological anchor against the rapid collapse of the null distribution demonstrates that ROB-Fold’s identified scaffolds are specifically linked to local biological relevance and are not trivial artifacts of background sequence homology.

The complete ROB-Fold pipeline was executed for each of the 50 non-biological anchors. The results of this test (Figure 12) reveal a stark contrast between biological and stochastic signals:

- Biological Motif (CXXC): Exhibits a sustained conservation curve, maintaining a similarity score above 50% even at expansion lengths exceeding 400 residues.
- Non-biological results: The full null distribution shows an immediate and consistent decay. While the biological anchors start at high similarity due to the forced anchoring, they fail to identify consistent evolutionary segments during expansion. Most non-biological trials collapse shortly after the seed, as they lack the functional and evolutionary pressure required to sustain a structural scaffold.

## 4 Discussion

### 4.1 Algorithmic adaptability and cross-family reproducibility

The rule-based nature of ROB-Fold inherently supports its transferability across protein families. Unlike data-driven algorithms that rely on learned embeddings or family-specific training datasets, ROB-Fold operates on structural logic, a set of explicit, user defined spatial and sequential rules that describe conserved regions within any protein architecture. This design allows the substitution of family-specific anchors (e.g., CXXC in PDIs) by any other structurally or functionally meaningful motif (e.g., EEVD in HSP70s), without requiring retraining or reparameterization of the algorithmic core.

Such modularity arises from the algorithm’s separation of concerns:

1. Motif detection layer, which identifies local anchor patterns based on regular expressions or structural fingerprints.
2. Expansion and overlap layer, which propagates conservation windows according to geometric constraints, independent of amino acid identity.
3. Consensus analysis layer, which integrates conservation frequency and RMSD based similarity to extract recurrent scaffolds.

Because these layers are parameterized through a configuration interface rather than hard coded to any biological family, the algorithm can be readily adapted to new systems with minimal modification.

### 4.2 ROB-fold powerful tracking of evolution against non-biological anchors

As shown in Figure 12, this null model of the non-biological anchor produces a distribution of conservation decay curves (gray curves), representing the expected behavior of structurally valid but non-functional positions. In contrast, the biological motif, the biological anchor (CXXC), consistently exhibits higher conservation across all expansion lengths (red curve) and lies outside the null distribution for most of the range.

From the earliest stages of expansion, the permuted sequences begin to rapidly lose structural conservation. At the initial point, when the analysis window is small, several gray curves still display relatively high similarity values, although objectively lower than the behavior of the biological anchor. However, as expansion increases, approximately between 40 and 100 residues, these curves drop markedly, indicating that the matched non-biological anchor motifs fail to sustain a stable conservation signal when additional structural context is incorporated around the anchor.

At intermediate expansions, approximately between 120 and 200 residues, most gray curves cluster at considerably lower conservation values. This behavior defines a basal level of structural stability, corresponding to cases where only amino acid composition and local structural environment are preserved, but not the biological organization of the anchor motif. In contrast, the red curve, corresponding to the biological anchor, remains consistently above this null distribution throughout the expansion. Although it shows a gradual decay as the window length increases, this decay is significantly smaller compared to the null model, indicating greater resistance to structural degradation.

The separation between the trajectory of the biological anchor and the distribution of gray curves clearly demonstrates that the null model remains at low similarity levels, while the biological motif retains a higher score. This sustained difference shows that the observed structural persistence does not arise from general sequence properties such as amino acid composition or structural context, but rather from a specific and non-random organization of residues.

Taken together, these results indicate that the structural conservation associated with the biological anchor is an intrinsic property of the sequential ordering of the anchor motif and cannot be reproduced by preserving only amino acid composition or structural environment.

### 4.3 Benchmarking ROB-Fold motif detection against MEME and PROSITE

The motif-detection accuracy of ROB-Fold was assessed against PROSITE across three functionally characterized anchor motifs: CXXC (PDI family), EEVD (HSP70 family), and YXR. Agreement was quantified using an Intersection-over-Union (IoU) threshold of ≥0.5 at the site level. The MEME-based comparison included in the initial submission has been removed; that evaluation yielded TP = 0 for all motifs owing to a systematic identifier and coordinate mismatch rather than a biological disagreement, and its results were therefore non-informative. PROSITE, which encodes the same class of rule-based patterns as ROB-Fold, constitutes the appropriate gold standard for this evaluation.

#### 4.3.1 PROSITE comparison results

The initial submission reported a CXXC F1-score of 0.920, reflecting two false-positive and two false-negative calls in the experimentally resolved PDI structure 2b5e. A code audit identified the cause: 2b5e contains a hetero-atom entry at PDB position 1 followed by the polypeptide chain beginning at residue 22, creating a 20-residue gap in the numbering index. The original implementation reported motif coordinates as 1-based positions within the extracted sequence string without translating them back to PDB residue numbers, placing the reported CXXC sites at positions 41–44 and 386–389 instead of the correct PDB residues 61–64 and 406–409. The pipeline was corrected by mapping each regex match index to the corresponding.

Residue number attribute of the parsed PDB object, ensuring that coordinates are always reported in PDB residue space regardless of whether the structure uses contiguous (AlphaFold) or non-contiguous (crystal) residue numbering.

Following this correction, ROB-Fold achieves perfect agreement with PROSITE across all three motif types (Table 4). For CXXC, 25 hits are detected by both methods, yielding 25 TP, 0 FP, and 0 FN (precision = recall = F1 = 1.000, mean IoU = 1.000). Results for EEVD (7 sites across 7 HSP70 proteins) and YXR (4 sites) were already perfect in the original submission and are unchanged. Micro-averaged across all three motifs, precision = recall = F1 = 1.000, confirming that ROB-Fold faithfully reproduces PROSITE annotations both in site detection and in boundary placement.

**TABLE 4:** Performance metrics for ROB-Fold vs. PROSITE. IoU overlap criterion ≥0.5. All coordinates reported in PDB residue space. CXXC improvement reflects the coordinate correction for crystal structures with non-contiguous PDB numbering.

Motif	Reference	ROB-fold hits	Reference hits	TP	FP	FN	Precision	Recall	F1	Mean IoU
CXXC	PROSITE	25	25	25	0	0	1.000	1.000	1.000	1.000
EEVD	PROSITE	7	7	7	0	0	1.000	1.000	1.000	1.000
YXR	PROSITE	4	4	4	0	0	1.000	1.000	1.000	1.000
**Micro-avg**	**—**	**36**	**36**	**36**	**0**	**0**	**1.000**	**1.000**	**1.000**	**1.000**

#### 4.3.2 Empirical justification of the 15 Å RMSD threshold

The 15 Å cutoff used to delineate structurally conserved scaffold regions is grounded in the observed pairwise RMSD distribution rather than set *a priori*. At the anchor motif itself (window length = 4 residues), mean RMSD is 0.37 Å for PDI and 0.43 Å for HSP70, confirming near-perfect superimposition at the functional core. As window length increases, RMSD grows progressively but remains tightly controlled within the scaffold boundary: 94.9% of PDI pairwise comparisons and 100% of HSP70 comparisons at windows up to 104 residues fall below 15 Å. Across all window lengths combined, 84.9% of PDI pairs and 99.1% of HSP70 pairs satisfy RMSD <15 Å.

Table 5 shows mean RMSD and the fraction of pairs below threshold at representative window lengths. A natural inflection is visible in both families: values remain well below 15 Å throughout windows of 4–104 residues and then rise sharply, reaching 14.16 Å (PDI) and 11.45 Å (HSP70) at 184 residues, before the fraction of compliant pairs drops steeply beyond 200 residues. This transition marks the boundary between the conserved scaffold core and the flexible, adaptive linker regions, providing a data-driven basis for the 15 Å cutoff reported in the main text.

**TABLE 5:** Mean RMSD (Å) and fraction of pairs below 15 Å by window length. RMSD from Cα-atom superimposition (Bio.PDB Superimposer). HSP70 pair counts decrease at longer windows because some AlphaFold models are shorter than the full expansion radius.

Window (res.)	PDI n	PDI mean RMSD (Å)	PDI <15 Å	HSP70 n	HSP70 mean RMSD (Å)	HSP70 < 15 Å
4	289	0.37	100.0%	21	0.43	100.0%
24	300	1.39	100.0%	21	1.76	100.0%
44	276	3.05	100.0%	21	3.47	100.0%
64	276	4.60	98.2%	21	4.40	100.0%
104	231	10.72	84.4%	21	7.62	100.0%
144	123	12.02	74.8%	21	9.13	100.0%
184	81	14.16	59.3%	21	11.45	100.0%
224	24	16.87	20.8%	21	13.22	100.0%
264	18	17.18	27.8%	15	14.80	86.7%
304	18	17.64	22.2%	9	15.23	77.8%

### 4.4 Computational efficiency and performance benchmarking

To evaluate the practical viability of ROB-FOLD in diverse research settings, we conducted a comprehensive computational performance benchmark across various hardware configurations. As summarized in Table 6, the algorithm’s efficiency was assessed by comparing execution times before and after a strategic code optimization phase. Our results demonstrate a substantial reduction in processing time, with the optimized version achieving sub-10-s execution for most protein datasets on consumer-grade hardware.

**TABLE 6:** Performance metrics for ROB-Fold.

CPU	Cores	Threads	Frequency [GHz]	RAM [GB]	Pre-optimization time [s]	Post-optimization time [s]
Intel CORE i5	4	8	2.5	8	633.37	8.67
AMD ryzen 3 7330U	4	8	2.3	16	750	14
AMD ryzen 5 8500G	6	12	3.5	16	524.13	7.78
AMD ryzen 5 5500	6	12	3.6	32	729.666	8.55

These findings confirm that ROB-Fold is a computationally sustainable tool for large-scale structural analysis, enabling researchers to perform complex bioinformatic workflows locally without the necessity for high-performance computing (HPC) infrastructure.

### 4.5 Future work

Despite the versatility demonstrated by ROB-Fold in the analysis of the PDI and HSP70 families, it is important to acknowledge the inherent limitations of rule-based approaches. First, the algorithm depends on the explicit definition of a small anchor motif or structural reference region. While this design ensures interpretability and experimental control, it also constrains its ability to discover completely novel motifs or unexpected conserved regions beyond predefined anchors.

A major future direction involves integrating machine learning modules capable of identifying previously unknown structural motifs, which could serve as new anchors for ROB-Fold’s rule-based inference engine. Conversely, the results generated by ROB-Fold including conservation matrices, consensus structures and geometric relationships can serve as structured and interpretable datasets for training generative models aimed at designing new proteins that preserve the topological and functional coherence of natural scaffolds.

In this way, ROB-Fold is envisioned not merely as a structural analysis tool but as a bidirectional framework linking symbolic reasoning with statistical intelligence: a system capable of learning from nature through explicit rules while simultaneously feeding artificial intelligence with interpretable knowledge for the rational creation of novel protein architectures.

ROB-Fold is an epistemically robust framework that derives its principles from the lessons of nature, advancing along an economically efficient and sustainable trajectory; grounded in abductive reasoning, it holds the potential—particularly through the integration of generative artificial intelligence—to design and synthesize novel proteins in a manner analogous to natural processes.

## Data Availability

The original contributions presented in the study are included in the article/supplementary material, further inquiries can be directed to the corresponding author.

## References

[B1] Al-Gharabli S. I. Al-Agtash S. Rawashdeh N. A. Barqawi K. R. Almagro Armenteros J. J. Sønderby C. K. (2018). DeepLoc: prediction of protein subcellular localization using deep learning. Bioinformatics 33, 41–49. 10.1093/bioinformatics/bty116 29028934

[B2] AlQuraishi M. (2021). Machine learning in protein structure prediction. Curr. Opin. Chem. Biol. 65, 1–8. 10.1016/j.cbpa.2021.04.005 34015749

[B3] Dara S. Dhamercherla S. Jadav S. S. Babu C. H. Ahsan M. J. Hie B. L. (2019). Prediction of protein metal binding sites using deep neural networks. Bioinformatics 35, 131–148. 10.1002/minf.201800169

[B4] Gribaldo S. Lumia V. Creti R. Conway De Macario E. Sanangelantoni A. Cammarano P. (1999). Discontinuous occurrence of the hsp70 (dnaK) gene among Archaea and sequence features of HSP70 suggest a novel outlook on phylogenies inferred from this protein. J. Bacteriology 181, 434–443. 10.1128/JB.181.2.434-443.1999 9882656 PMC93396

[B5] Hey T. Butler K. Jackson S. Thiyagalingam J. (2019). Machine learning and big scientific data. Philos. Trans. A Math. Phys. Eng. Sci. 378, 10.1098/RSTA.2019.0054 31955675 PMC7015290

[B6] Kampinga H. H. Craig E. A. (2010). The HSP70 chaperone machinery: j proteins as drivers of functional specificity. Nat. Reviews. Mol. Cell Biology 11, 579–592. 10.1038/NRM2941 20651708 PMC3003299

[B7] Kayum M. A. Park J. I. Nath U. K. Saha G. Biswas M. K. Kim H. T. (2017). Genome-wide characterization and expression profiling of PDI family gene reveals function as abiotic and biotic stress tolerance in Chinese cabbage (Brassica rapa ssp. pekinensis). BMC Genomics 18, 885. 10.1186/s12864-017-4277-2 29145809 PMC5691835

[B8] Kim J.-H. Zhao Y. Pan X. He X. Gilbert H. F. (n.d.). The unfolded protein response is necessary but not sufficient to compensate for defects in disulfide isomerization. J. Biol. Chem. 284, 10496–10506. 10.1074/jbc.M900377200 19233841 PMC2667727

[B9] Kroth H. Ansaloni A. Varisco Y. Jan A. Sreenivasachary N. Rezaei-Ghaleh N. (2012). Discovery and structure activity relationship of small molecule inhibitors of toxic $β$-amyloid-42 fibril formation. J. Biol. Chem. 287, 34786–34800. 10.1074/jbc.M112.357665 22891248 PMC3464581

[B10] Liang M. Nie J. (2020). Prediction of enzyme function based on a structure relation network. IEEE ACCESS 8, 132360–132366. 10.1109/ACCESS.2020.3010028

[B11] Moretti A. I. S. Pavanelli J. C. Nolasco P. Leisegang M. S. Tanaka L. Y. Fernandes C. G. (2017). Conserved gene microsynteny unveils functional interaction between protein disulfide isomerase and rho guanine-dissociation inhibitor families. Sci. Rep. 7, 17262. 10.1038/s41598-017-16947-5 29222525 PMC5722932

[B12] Qin Z. Wu L. Sun H. Huo S. Ma T. Lim E. (2020). Artificial intelligence method to design and fold alpha -helical structural proteins from the primary amino acid sequence. EXTREME Mech. Lett. 36, 100652. 10.1016/j.eml.2020.100652

[B13] Raveh B. Basri R. Schreiber G. (2007). Rediscovering secondary structures as network motifs–an unsupervised learning approach. Bioinformatics 23. 10.1093/bioinformatics/btl290 17237086

[B14] Senior A. W. Evans R. Jumper J. Kirkpatrick J. Sifre L. Green T. (2020). Improved protein structure prediction using potentials from deep learning. Nature 577, 706–710. 10.1038/s41586-019-1923-7 31942072

[B15] Sharma D. Masison D. (2009). Hsp70 structure, function, regulation and influence on yeast prions. Protein Peptide Letters 16, 571–581. 10.2174/092986609788490230 19519514 PMC2746719

[B16] Sharma D. Martineau C. N. Le Dall M. T. Reidy M. Masison D. C. Kabani M. (2009). Function of SSA subfamily of Hsp70 within and across species varies widely in complementing *Saccharomyces cerevisiae* cell growth and prion propagation. PloS One 4, e6644. 10.1371/JOURNAL.PONE.0006644 19680550 PMC2721632

[B17] Siedhoff N. E. Schwaneberg U. Davari M. D. (2020). Machine learning-assisted enzyme engineering. 1st Edn. Elsevier Inc. 10.1016/bs.mie.2020.05.005 32896285

[B18] Villalobos-Alva J. Ochoa-Toledo L. Villalobos-Alva M. J. Aliseda A. Pérez-Escamirosa F. Altamirano-Bustamante N. F. (2022). Protein science meets artificial intelligence: a systematic review and a biochemical meta-analysis of an inter-field. Front. Bioeng. Biotechnol. 10, 788300. 10.3389/fbioe.2022.788300 35875501 PMC9301016

[B19] Westphal V. Spetzler J. C. Meldal M. Christensen U. Winther J. R. (n.d.). Kinetic analysis of the mechanism and specificity of protein-disulfide isomerase using fluorescence-quenched pep-tides. J. Biol. Chem. 273, 24992–24999. 10.1074/jbc.273.39.24992 9737954

[B20] Yamada K. D. Kinoshita K. (2018). *De novo* profile generation based on sequence context specificity with the long short-term memory network. BMC Bioinforma. 19, 272. 10.1186/s12859-018-2284-1 30021530 PMC6052547

[B21] Yang J. Shen C. Huang N. (2020). Predicting or pretending: artificial intelligence for protein-ligand interactions lack of sufficiently large and unbiased datasets. Front. Pharmacol. 11, 1–9. 10.3389/fphar.2020.00069 32161539 PMC7052818

